# A bibliometric analysis of respiratory mechanics research related to acute respiratory distress syndrome from 1985 to 2023

**DOI:** 10.3389/fmed.2024.1420875

**Published:** 2024-09-20

**Authors:** Yi-Min Zhou, Xiuli Tian, Yu-Mei Wang, Shuya Wang, Yan-Lin Yang, Jian-Xin Zhou, Linlin Zhang

**Affiliations:** ^1^Department of Critical Care Medicine, Beijing Tiantan Hospital, Capital Medical University, Beijing, China; ^2^Department of Respiration, Liaocheng People’s Hospital, Liaocheng, China; ^3^Department of Critical Care Medicine, Beijing Shijitan Hospital, Capital Medical University, Beijing, China

**Keywords:** acute respiratory distress syndrome, respiratory mechanics, mechanical ventilation, bibliometric analysis, international collaboration

## Abstract

**Background:**

Acute respiratory distress syndrome (ARDS) is a severe condition characterized by lung stiffness and compromised gas exchange, often requiring mechanical ventilation for treatment. In addition to its clinical significance, understanding the publication trends and research patterns in respiratory mechanics related to ARDS can provide insights into the evolution of this field from a bibliometric perspective, aiding in strategic planning and resource allocation for future research endeavors.

**Objective:**

This study aimed to explore the trends and identify the hotspots in respiratory mechanics research related to ARDS.

**Methods:**

All relevant studies on respiratory mechanics of ARDS published between 1985 and 2023 were retrieved from the Web of Science Core Collection (WoSCC), and the retrieval strategy was topic search “TS = respiratory mechanics OR lung mechanics AND TS = ARDS OR acute respiratory distress syndrome.” Annual trends, citation patterns, and contributions from countries, institutions, authors, and journals were analyzed using Bibliometrix Biblioshiny. Networks and overlay of authors, institutions, countries, journals, co-citations, and keywords were analyzed and visualized using VOSviewer.

**Results:**

Our analysis included 1,248 articles published between 1985 and 2023, revealing fluctuations in publication output over time. The United States emerged as the leading contributor, with *Critical Care Medicine* being the most prominent journal. Key research themes included mechanical ventilation, acute lung injury, and protective ventilation strategies. International collaboration was evident, facilitating knowledge exchange and interdisciplinary cooperation.

**Conclusion:**

Our study sheds light on the evolving landscape of respiratory mechanics research in ARDS. International collaboration is pivotal in advancing the field, while researchers increasingly focus on personalized approaches to address the complexities of ARDS respiratory mechanics.

## Introduction

Since its first description in 1967, acute respiratory distress syndrome (ARDS), characterized by stiff lungs and a “baby lung”, has been researched for over 55 years (1, 2). Over the past five decades, several milestones have shaped our understanding and treatment of ARDS (3). Notably, the introduction of the American–European Consensus Conference definition in 1994 standardized the diagnosis and significantly influenced clinical trials and practice. The subsequent Berlin Definition in 2012 further refined ARDS classification, leading to a resurgence in research focused on this syndrome (4). The new global definition of ARDS proposed in 2024 further expands the definition of ARDS, promoting its early recognition and diagnosis and ensuring that patients with ARDS in resource-limited areas are no longer excluded (5). Despite these advancements, the mainstay of therapy remains mechanical ventilation, which, while life-saving, can also cause iatrogenic lung injury if not carefully managed (6–8). The recognition of ventilator-induced lung injury (VILI), the development of lung-protective strategies, such as the use of low tidal volumes and appropriate positive end-expiratory pressure (PEEP), and prone position ventilation represent critical advancements in ARDS management (9–11). In this scenario, respiratory mechanics plays an essential role in ARDS management. First, mechanics variables such as respiratory system compliance, driving pressure, and mechanical power were considered markers of the severity of ARDS and contributed to a better understanding of its pathogenesis (12, 13). Second, respiratory mechanical parameters can guide individualized mechanical ventilation therapy in ARDS patients, reducing the risk of ventilator-associated lung injury and enabling the evaluation of therapeutic effects through bedside monitoring (14–16).

The widespread application and popularity of the Internet have led to an explosive increase in the publication and citation volume of medical literature (17). Bibliometric analysis, a well-established and widely recognized research method, is frequently used to quantify the impact of scientific articles and evaluate contributions to a research field, including those by countries, institutions, authors, and journals (18). Through information visualization and bibliometric analysis, this method can also reveal trends in temporal changes and research hotspots in a specific research field (19, 20). At present, it has been widely used in medical research on sepsis (21), nursing (22), and stomatology (23). Despite the existence of a limited number of bibliometric analysis studies in the field of ARDS, to our knowledge, no bibliometric studies have focused explicitly on ARDS’s respiratory mechanics (24–26). Therefore, our study fills this gap and offers a unique perspective on the field.

In this study, we conducted an exhaustive bibliometric analysis of literature on ARDS-related respiratory mechanics, encompassing the annual number of publications, countries, journals, authors, international cooperation, and institutions analysis. Furthermore, overlay visualization maps of co-occurring keywords and clustering analysis were performed to identify the trends and hotspots of respiratory mechanics research related to ARDS. This study offers a new perspective and serves as a foundation for future research on ARDS-related respiratory mechanics.

## Methods

### Data sources and search strategies

All data for this study were retrieved from the Web of Science Core Collection (WoSCC) via the Capital Medical University Library website. The retrieval strategy was “[TS = (respiratory mechanics OR lung mechanics)] AND [TS = (ARDS OR acute respiratory distress syndrome)].” The publication period was between 1985 and 2023. Publications in languages other than English were excluded. All searches were conducted on 6 June 2024 to avoid bias in database updates. WoSCC data, including titles, authors, institutions, countries, journals, abstracts, keywords, and references, were downloaded in plain TXT format. Data collection was performed online, and ethical approval was not required. Two investigators (ZYM and WYM) independently performed the searches, demonstrating significant consistency (*κ* = 97%).

### Bibliometric analysis and visualization

The raw data were imported to Bibliometrix Biblioshiny, an online platform of literature metrology, for bibliometric analysis (27). VOSviewer 1.6.19 (Leiden University, Leiden, The Netherlands) was used to analyze and visualize networks and overlay of authors, institutions, countries, journals, co-citations, and keywords (28).

## Results

**Figure 1:**
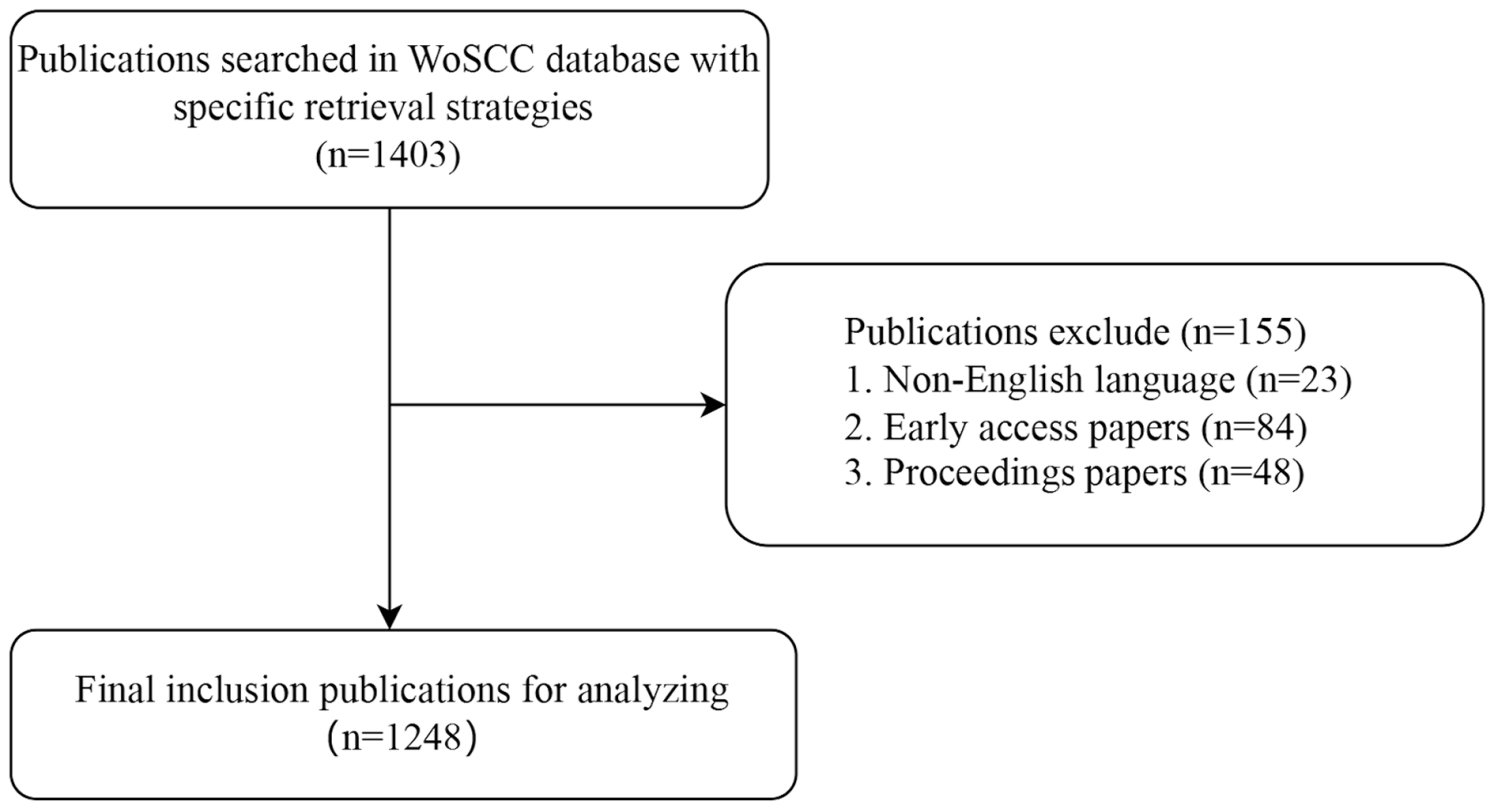
Flowchart of publications including and excluding. WoSCC, Web of Science Core Collection.

A total of 1,403 articles related to respiratory mechanics of ARDS published between 1985 and 2023 were initially identified. After excluding 23 non-English-language publications, 84 early access articles, and 48 proceedings articles, 1,248 publications were ultimately analyzed (Figure 1).

### Annual tendencies and citations

**Figure 2:**
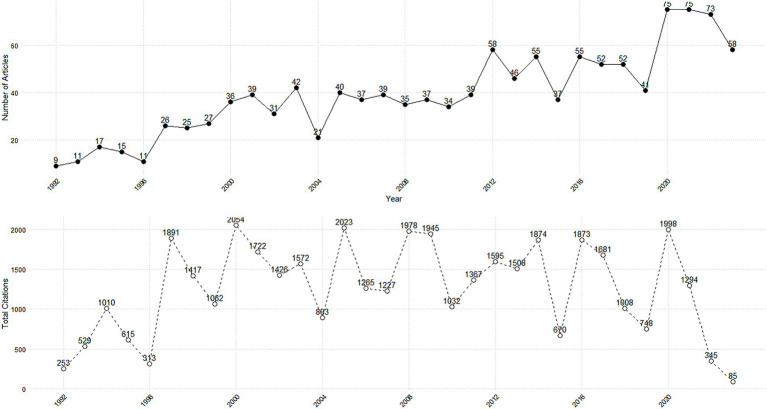
Annual trends in publications (upper panel) and citations (bottom panel) related to the respiratory mechanics of ARDS.

Figure 2 illustrates the annual trends in publications related to the respiratory mechanics of ARDS. The number of articles published each year varied, with notable peaks observed in 2020 and 2021, during which 75 articles were published each year, constituting 6.0% of the total publications. The total number of articles cited during the entire period covered by the data was 40,272, with an average citation count of 32.27 per article. From 1992 to 2005, the number of articles remained relatively stable, followed by a significant increase, especially between 2012 and 2021.

### Contribution of countries and institutions

Between 1985 and 2023, 45 countries or regions and 1,316 institutions contributed to publications on the respiratory mechanics of ARDS. The top 10 countries or regions and the top 10 institutions are presented in Tables 1, 2. The United States emerged as the most significant contributor, with 296 articles published, followed by Italy (*n* = 165), Germany (*n* = 115), China (*n* = 88), France (*n* = 79), Brazil (*n* = 62), Canada (*n* = 58), Sweden (*n* = 39), Spain (*n* = 36), and Greece (*n* = 26). The Federal University of Rio de Janeiro led in productivity with 126 articles published, followed by the University of Milan (*n* = 112), University of Toronto (*n* = 99), University of São Paulo (*n* = 86), Harvard University (*n* = 59), University of Genoa (*n* = 52), University of California, San Francisco (*n* = 49), Uppsala University (*n* = 43), Massachusetts General Hospital (*n* = 40), and University of Minnesota (*n* = 37).

**Table 1:** The top 10 countries/regions contributing to publications on respiratory mechanics of ARDS.

Rank	Country/region	Articles	Percentage (%)	TC	Average article citations
1	United States	296	23.7	10,431	35.20
2	Italy	165	13.2	7,596	46.00
3	Germany	115	9.2	3,877	33.70
4	China	88	7.1	717	8.10
5	France	79	6.3	2,808	35.50
6	Brazil	62	5	2,260	36.50
7	Canada	58	4.6	3,125	53.90
8	Sweden	39	3.1	1,143	29.30
9	Spain	36	2.9	821	22.80
10	Greece	26	2.1	510	19.60

ARDS, acute respiratory distress syndrome; TC, total citations.

**Table 2:** The top 10 affiliations contributing to publications on respiratory mechanics of ARDS.

Rank	Affiliations	Country	Articles	TC	Number of first author	TC of the first author
1	Federal University of Rio de Janeiro	Brazil	126	2,151	39	1,549
2	University of Milan	Italy	112	5,722	46	2,400
3	University of Toronto	Canada	99	4,583	11	570
4	University of São Paulo	Brazil	86	2,546	12	488
5	Harvard University	United States	59	1800	10	253
6	University of Genoa	Italy	52	1,224	14	231
7	University of California San Francisco	United States	49	1,222	10	392
8	Uppsala University	Sweden	43	754	14	253
9	Massachusetts Gen Hosp	United States	40	1,615	16	547
10	University of Minnesota	United States	37	1,227	15	378

ARDS, acute respiratory distress syndrome; TC, total citations.

**Figure 3:**
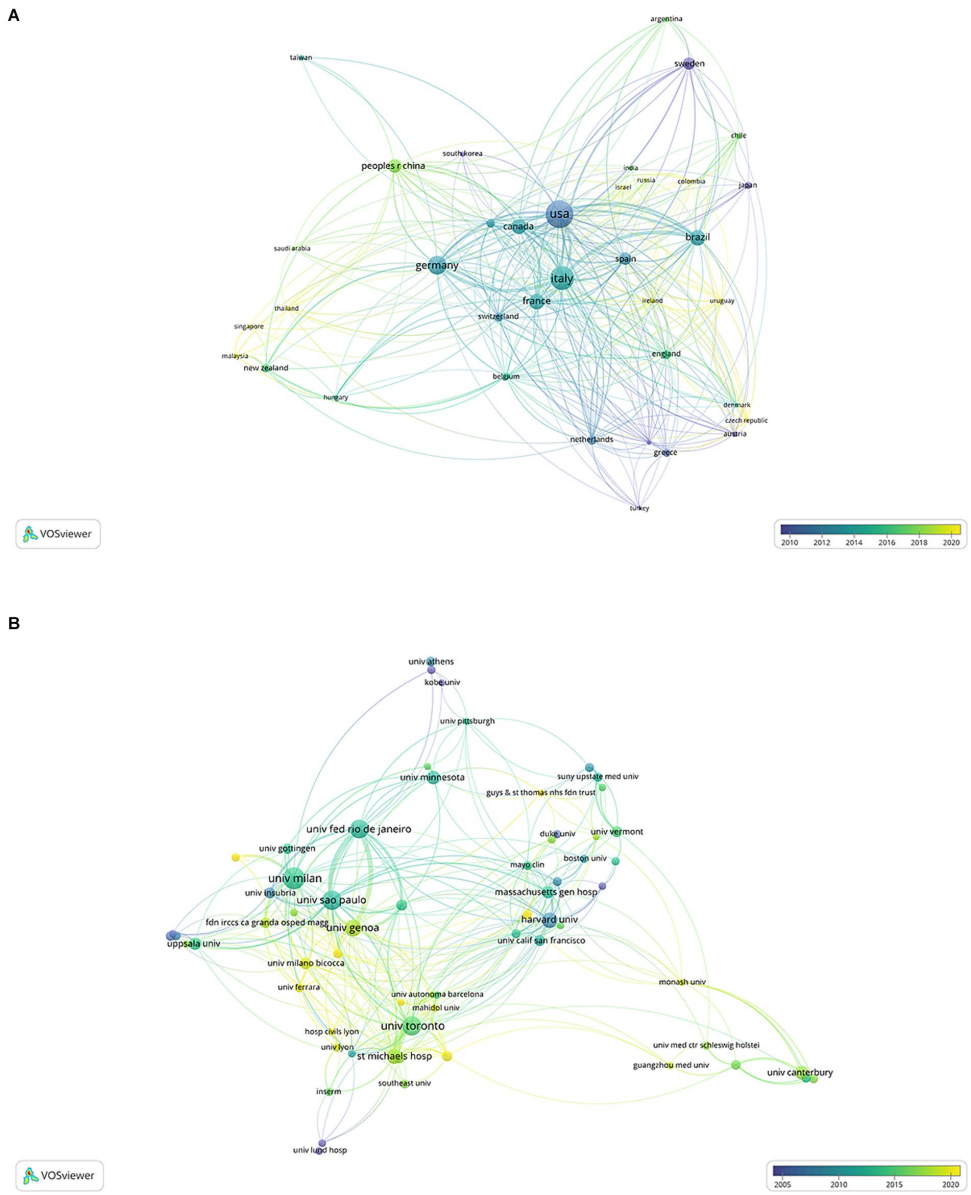
Co-authorship overlay visualization map of countries **(A)** and institutions **(B)**. The color of each circle corresponds to the average publication year, the size of a circle is proportional to the volume of literature, and the thickness of the connecting line indicates the cooperation frequency.

VOSviewer software generated a collaboration network based on countries and institutions. As depicted in Figures 3A,B, close cooperation among research institutions from various countries was observed during the research period. The international cooperation analysis revealed that the United States was the most frequently involved in global collaborations, while the University of Milan was the most commonly engaged institution in international partnerships.

### High-yielding journals and the most influential articles

**Figure 4:**
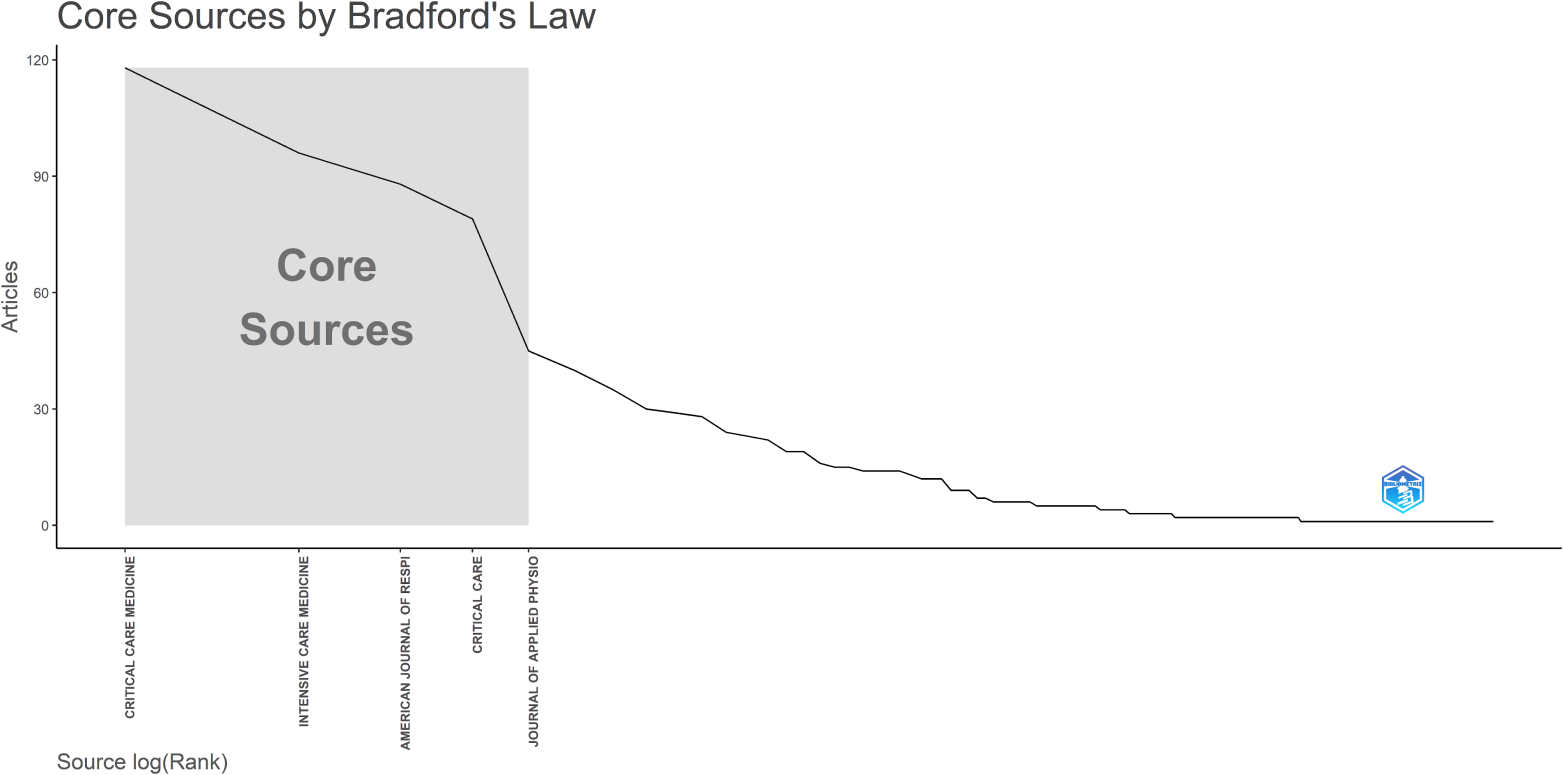
Journals in the core field according to Bradford’s law.

Until 31 December 2023, 1,248 publications on research linked to respiratory mechanics of ARDS were published in 235 journals, of which 48 contained at least 5 publications. The top 10 high-yielding journals are listed in Table 3, along with their publication counts, total citations, Hirsch index (h-index), Impact Factor in 2022 (IF 2022), and quartile in the category in 2022 (JCR 2022). *Critical Care Medicine* is the most prolific journal in this field with 118 publications, although its IF 2022, h-index, and total citations are not the highest. *Intensive Care Medicine* (*n* = 96) has the highest IF in 2022, and the *American Journal of Respiratory and Critical Care Medicine* (*n* = 88) has the highest h-index among the top 10 high-yielding journals. Other notable journals included *Critical Care* (*n* = 79), *Journal of Applied Physiology* (*n* = 45), *Respiratory Care* (*n* = 40), *European Respiratory Journal* (*n* = 35), *Current Opinion in Critical Care* (*n* = 30), *Annals of Intensive Care* (*n* = 29), and *Anesthesiology* (*n* = 28). According to Bradford’s law, five journals were in the core field (Figure 4). The top 10 most highly cited publications are listed in Table 4.

**Table 3:** The top 10 high-yielding journals in research on respiratory mechanics of ARDS.

Ranks	Journal title	Articles	TC	H-index	IF (2022)	JCR (2022)
1	Critical Care Medicine	118	4,966	41	8.8	Q1
2	Intensive Care Medicine	96	4,371	36	38.9	Q1
3	American Journal of Respiratory and Critical Care Medicine	88	7,918	50	24.7	Q1
4	Critical Care	79	2,603	31	15.1	Q1
5	Journal of Applied Physiology	45	1,485	22	3.3	Q2
6	Respiratory Care	40	663	13	2.8	Q4
7	European Respiratory Journal	35	1,144	19	24.9	Q1
8	Current Opinion in Critical Care	30	731	17	3.3	Q2
9	Annals of Intensive Care	29	680	18	8.1	Q1
10	Anesthesiology	28	1753	21	8.8	Q1

ARDS, acute respiratory distress syndrome; h-index, Hirsch index; IF, impact factor; JCR, journal citation report; TC, total citations.

**Table 4:** The top 10 most highly cited publications in research on respiratory mechanics of ARDS.

Rank	Title	Authors	Years	Journal	TC	TC per year
1	Acute respiratory distress syndrome caused by pulmonary and extrapulmonary disease different syndromes?	Gattinoni et al.	1998	American Journal of Respiratory and Critical Care Medicine	582	21.56
2	The effects of ibuprofen on the physiology and survival of patients with sepsis.	Bernard et al.	1997	New England Journal of Medicine	575	20.54
3	Lung stress and strain during mechanical ventilation for acute respiratory distress syndrome.	Chiumello et al.	2008	American Journal of Respiratory and Critical Care Medicine	489	28.76
4	Ventilator-related causes of lung injury: the mechanical power	Gattinoni et al.	2016	Intensive Care Medicine	456	50.67
5	Current perspectives in pulmonary surfactant—Inhibition, enhancement and evaluation	Zuo et al.	2008	Biochimica et Biophysica Acta – Bioenergetics	405	23.82
6	Lower tidal volume strategy (≈3 mL/kg) combined with extracorporeal CO2 removal versus ‘conventional’ protective ventilation (6 mL/kg) in severe ARDS	Bein et al.	2013	Intensive Care Medicine	382	31.83
7	Tidal volume reduction in patients with acute lung injury when plateau pressures are not high	Hager et al.	2015	American Journal of Respiratory and Critical Care Medicine	372	18.60
8	Contemporary management of acute right ventricular failure: a statement from the Heart Failure Association and the Working Group on Pulmonary Circulation and Right Ventricular Function of the European Society of Cardiology	Harjola et al.	2016	European Journal of Heart Failure	365	40.56
9	The application of esophageal pressure measurement in patients with respiratory failure	Akoumianaki et al.	2014	American Journal of Respiratory and Critical Care Medicine	349	31.73
10	Effects of recruiting maneuvers in patients with acute respiratory distress syndrome ventilated with protective ventilatory strategy	Grasso et al.	2002	Anesthesiology	317	13.78

ARDS, acute respiratory distress syndrome; TC, total citations.

### Contribution of authors

The top 10 most productive authors in respiratory mechanics research related to ARDS are presented in Table 5. Pelosi P from the Department of Anesthesia and Intensive Care, San Martino Policlinico Hospital in Italy, ranks first with 78 articles published and has the highest h-index. The top 3 authors with the most citations are Gattinoni L, Pelosi P, and Brochard L (4,567 vs. Four,360 vs. Two,489 times, respectively, Table 5).

**Table 5:** The top 10 most productive authors in the field link to respiratory mechanics of ARDS.

Rank	Author	Articles	TC	H-index
1	Pelosi P.	78	4,360	33
2	Rocco P.R.M.	51	1,594	23
3	Gattinoni L.	45	4,567	28
4	Chiumello D.	37	2,483	21
5	Capelozzi V.I.	35	1,386	21
6	Brochard L.	31	2,489	21
7	Chase J.G.	25	521	16
8	Marini J.J.	25	1,206	12
9	Hedenstierna G.	23	1,047	14
10	Guttmann J.	22	660	16

ARDS, acute respiratory distress syndrome; TC, total citations.

**Figure 5:**
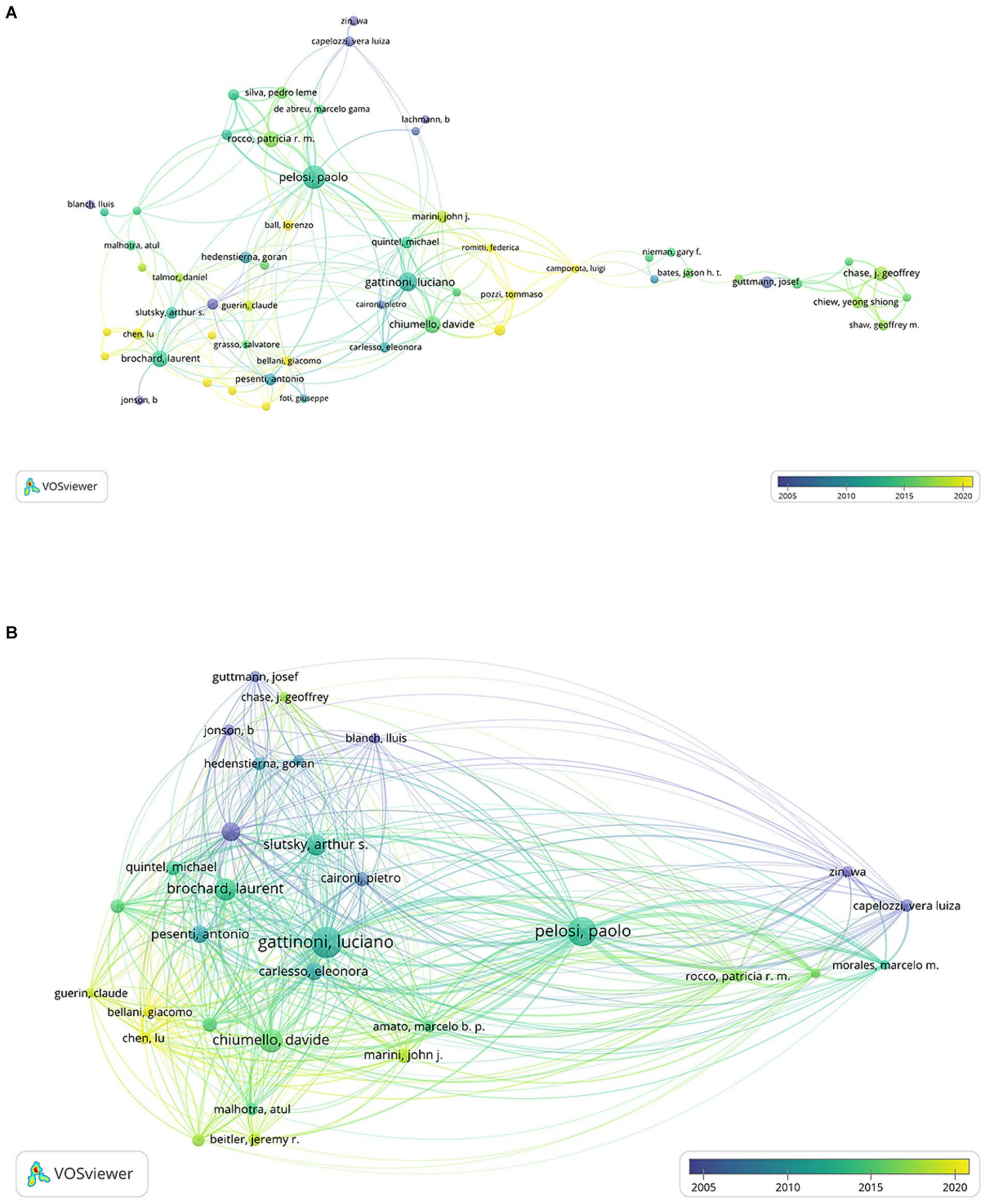
The distribution of authors in respiratory mechanics of ARDS research. **(A)** Co-authorship overlay visualization map of authors. The color of each circle corresponds to the average publication year of the author, the size of a circle is proportional to the number of literature, and the thickness of the connecting line indicates the strength of the co-occurrence link. **(B)** Co-citation overlay visualization map of authors. The color of each circle corresponds to the average publication year of the author, the size of a circle is proportional to the total number of citations of the author, and the thickness of the connecting line indicates the strength of the co-citation.

A co-authorship overlay visualization map was generated using VOSviewer software, with a threshold set to a minimum of 10 documents per author. Finally, 62 authors who met the threshold were identified. As shown in Figure 5A, Pelosi P has the broadest range of collaborations, with a total of 25 links, followed by Gattinoni L (*n* = 19), Chiumello D (*n* = 19), and Brochard L (*n* = 17). A co-citation overlay visualization map was also generated, with the threshold set to a minimum of 500 citations per author. Finally, 34 authors who met the threshold were identified. It is evident from the visualization that Pelosi P and Gattinoni L have made significant contributions to respiratory mechanics in ARDS (Figure 5B).

### Analysis of keywords

**Figure 6:**
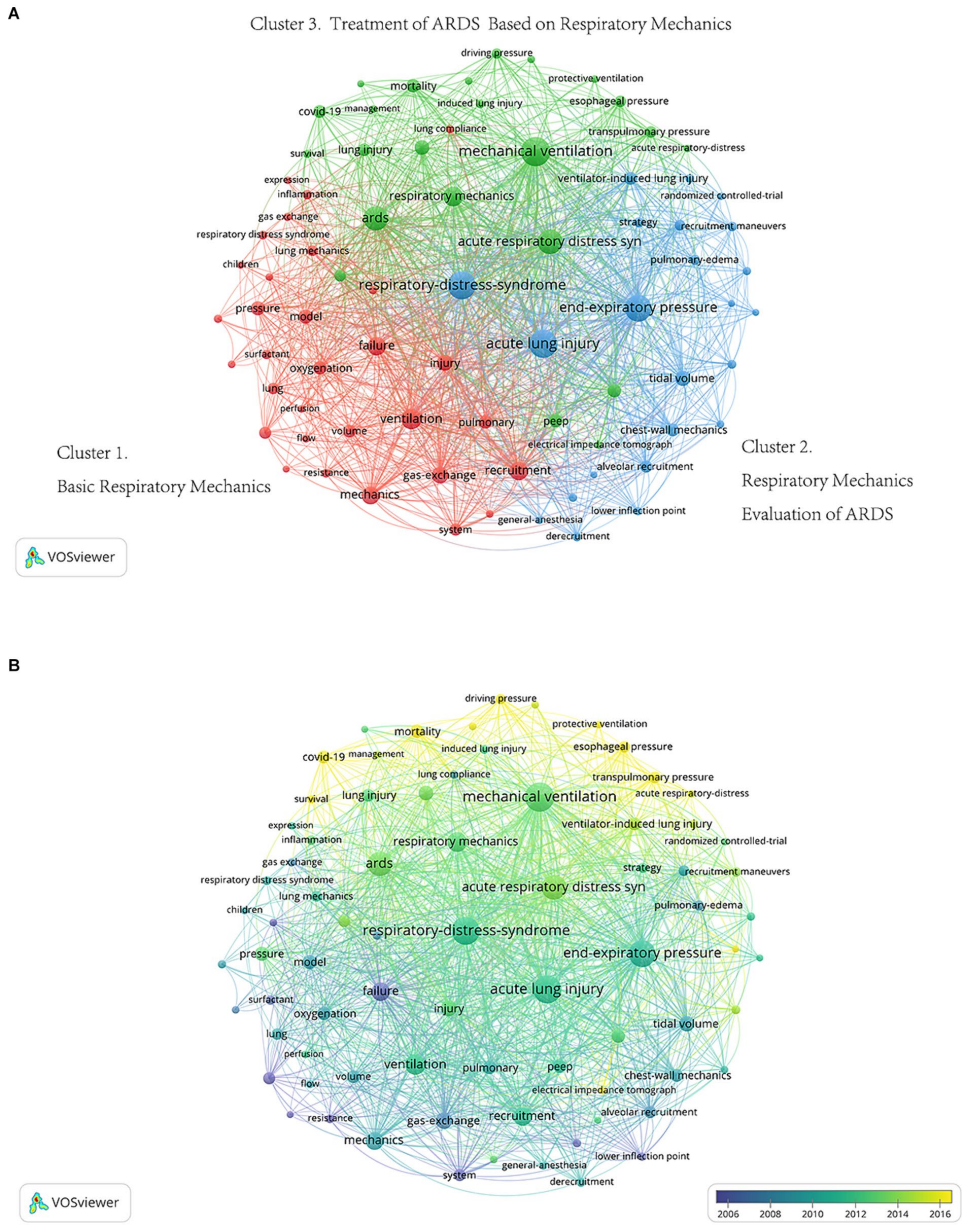
The co-occurrence map of keywords. **(A)** Co-occurrence network visualization map of keywords. Keywords were divided into three groups: basic respiratory mechanics (bottom left, red), respiratory mechanics evaluation of ARDS (bottom right, blue), and treatment of ARDS based on respiratory mechanics (top, green). **(B)** Co-occurrence overlay visualization map of keywords. The color of each circle corresponds to the average publication year of the author, the size of a circle is proportional to the total number of occurrences of the keyword, and the thickness of the connecting line indicates the strength of the co-occurrence link.

Co-occurrence analysis of keywords was performed with a minimum threshold of 30 occurrences. Ultimately, 77 keywords out of 3,783 keywords met the threshold and were divided into three groups: cluster 1, basic respiratory mechanics (bottom left, red); cluster 2, respiratory mechanics evaluation of ARDS (bottom right, blue); and cluster 3, treatment of ARDS based on respiratory mechanics (top, green, Figure 6A). The top 5 highest occurrence keywords were mechanical ventilation (cluster 3, 519 times), acute lung injury (cluster 2, 474 times), respiratory-distress-syndrome (cluster 2, 467 times), end-expiratory pressure (cluster 2, 421 times), and acute respiratory distress syndrome (cluster 3, 355 times). Detailed information on keywords is shown in Supplementary Table S1.

The co-occurrence overlay visualization map of keywords is shown in Figure 6B. Keywords such as coronavirus disease 2019 (COVID-19) (cluster 3, average publication year 2021), driving pressure (cluster 3, average publication year 2020), protective ventilation (cluster 3, average publication year 2017), transpulmonary pressure (cluster 3, average publication year 2017), electrical impedance tomography (cluster 3, average publication year 2016), and esophageal pressure (cluster 3, average publication year 2016) were the earliest keywords. Based on the color intensity, we found that the research hotspot in ARDS respiratory mechanics research, starting from basic respiratory mechanics, transitioning gradually to the assessment of respiratory mechanics in ARDS, and ultimately shifting toward the treatment of ARDS guided by respiratory mechanics.

## Discussion

In the present study, we conducted an exhaustive bibliometric analysis of respiratory mechanics research literature related to ARDS, leveraging the strengths of bibliometric methods to elucidate publication trends, research contributions, and emerging hotspots in the field. By harnessing the power of bibliometric analysis, we aim to provide valuable insights that can inform future research directions and clinical practice in the management of ARDS.

The observed fluctuations in the number of publications over time reflect the dynamic nature of research activity in respiratory mechanics and ARDS. While the number of publications remained relatively stable from 1992 to 2005, research output significantly increased, particularly between 2012 and 2021. The observed increase in publications around 2012 May be attributed to the introduction of the Berlin Definition of ARDS, which aimed to enhance clinical practice and research by offering clear criteria for ARDS diagnosis and classification, leading to an upsurge in scholarly activity focused on ARDS (4). The significant increase in publications observed around 2020 is likely attributed to the global COVID-19 pandemic (29, 30). The unprecedented public health crisis sparked by the emergence of the novel coronavirus led to an intensified focus on respiratory mechanics related to ARDS (31–34).

The dominance of certain countries, such as the United States, Italy, and Germany, in publication output, underscores their significant contributions to the field. The United States has been at the forefront, leading in innovative clinical trials and the development of advanced ventilatory strategies for ARDS management, with the establishment of the ARDS network likely making a significant contribution (9, 10). Italian researchers have made significant strides in understanding the pathophysiology of ARDS, with authors Pelosi P and Chiumello D making particularly outstanding contributions (35, 36). Over the past decade, China has been experiencing a notable increase in its publication output in this domain, mirroring the overall rise in SCI publications in critical care medicine originating from the country (37). Moreover, prolific authors such as Pelosi P and institutions such as the Federal University of Rio de Janeiro have played critical roles in shaping ARDS research through their extensive publications and collaborations. Pelosi P’s influential studies differentiate pulmonary from extrapulmonary ARDS. His research has been fundamental in understanding the unique characteristics and responses to treatment in these patient populations (35). Additionally, his investigations into the effects of prone positioning have provided a deeper understanding of how this technique can improve outcomes for patients with ARDS, underscoring the importance of our focus on respiratory mechanics in our current study (38, 39). The networks among countries and institutions highlight that international collaboration is crucial for advancing ARDS research. It facilitates knowledge exchange and fosters interdisciplinary cooperation (40).

In examining the contributions of journals to respiratory mechanic research related to ARDS, we observe significant variations in publication output and impact. Journals such as *Critical Care Medicine*, *Intensive Care Medicine*, and the *American Journal of Respiratory and Critical Care Medicine* emerge as prominent platforms for disseminating research findings, indicating their pivotal roles in facilitating scholarly discourse and knowledge dissemination. In respiratory mechanics research related to ARDS, the *American Journal of Respiratory and Critical Care Medicine* typically features studies that concentrate on the underlying pathophysiological mechanisms, associated complications, and the strategic management of patients. The *Critical Care Medicine* journal tends to present a more comprehensive array of topics, including various treatment approaches, the physiological impacts of the disease, and the general conditions of the illness. In contrast, the *Intensive Care Medicine* journal often prioritizes in-depth examinations of targeted interventions and the mechanisms at play. Understanding the landscape of journals in respiratory mechanic research related to ARDS is essential for researchers to strategically select suitable publication venues that align with their study objectives and target audiences, ultimately contributing to advancing knowledge in this critical area of medicine.

The co-occurrence analysis of keywords reveals distinct research themes and hotspots within respiratory mechanics research related to ARDS. These include fundamental concepts such as mechanical ventilation and acute lung injury, as well as emerging topics such as COVID-19-related respiratory mechanics and advanced monitoring techniques such as transpulmonary pressure and electrical impedance tomography. Visualizing keyword networks highlights the progression of research focused on basic respiratory mechanics, evaluating and treating ARDS by respiratory mechanics. The co-occurrence overlay visualization map of keywords indicates that researchers’ interest in the respiratory mechanics of ARDS is gradually shifting from basic understanding and treatment of the disease to more detailed and personalized research directions, aiming to understand better and address the complex respiratory mechanics features of ARDS (41, 42).

While this study provides valuable insights into the landscape of respiratory mechanics research related to ARDS, it has some limitations. First, the bibliometric analysis relies solely on data from the WoSCC, which May not encompass all relevant publications in the field. Other databases or sources, such as PubMed or Scopus, could provide additional data not captured in this analysis. Second, the inclusion criteria for selecting articles May introduce bias, as publications in non-English languages were excluded, potentially overlooking valuable research from non-English speaking countries. Furthermore, while co-occurrence analysis provides insights into research trends and hotspots, it does not necessarily imply causation or the true significance of the identified themes.

## Conclusion

Over time, there has been a growing focus on respiratory mechanics research related to ARDS, highlighting key topics such as mechanical ventilation and acute lung injury. This study provides valuable insights for future research and holds promise for advancing the treatment and management of ARDS patients.

## Data Availability

The original contributions presented in the study are included in the article/Supplementary material, further inquiries can be directed to the corresponding authors.

## References

[1] Ashbaugh DG Bigelow DB Petty TL Levine BE. Acute respiratory distress in adults. Lancet. (1967) 290:319–23. doi: 10.1016/s0140-6736(67)90168-7 4143721

[2] Gattinoni L Pesenti A. The concept of “baby lung”. Intensive Care Med. (2005) 31:776–84. doi: 10.1007/s00134-005-2627-z, PMID: 15812622

[3] Sahetya SK Mancebo J Brower RG. Fifty years of research in ARDS. Vt selection in acute respiratory distress syndrome. Am J Respir Crit Care Med. (2017) 196:1519–25. doi: 10.1164/rccm.201708-1629CI, PMID: 28930639 PMC5754449

[4] Ranieri VM Rubenfeld GD Thompson BT Ferguson ND Caldwell E Fan E. Acute respiratory distress syndrome: the Berlin definition. JAMA. (2012) 307:2526–33. doi: 10.1001/jama.2012.5669 22797452

[5] Matthay MA Arabi Y Arroliga AC Bernard G Bersten AD Brochard LJ. A new global definition of acute respiratory distress syndrome. Am J Respir Crit Care Med. (2024) 209:37–47. doi: 10.1164/rccm.202303-0558WS, PMID: 37487152 PMC10870872

[6] Slutsky AS Ranieri VM. Ventilator-induced lung injury. N Engl J Med. (2013) 369:2126–36. doi: 10.1056/NEJMra1208707 24283226

[7] Katira BH. Ventilator-induced lung injury: classic and novel concepts. Respir Care. (2019) 64:629–37. doi: 10.4187/respcare.07055, PMID: 31110032

[8] Brochard L Slutsky A Pesenti A. Mechanical ventilation to minimize progression of lung injury in acute respiratory failure. Am J Respir Crit Care Med. (2017) 195:438–42. doi: 10.1164/rccm.201605-1081CP, PMID: 27626833

[9] Brower RG Matthay MA Morris A Schoenfeld D Thompson BT Wheeler A. Ventilation with lower tidal volumes as compared with traditional tidal volumes for acute lung injury and the acute respiratory distress syndrome. N Engl J Med. (2000) 342:1301–8. doi: 10.1056/nejm200005043421801 10793162

[10] Brower RG Lanken PN MacIntyre N Matthay MA Morris A Ancukiewicz M. Higher versus lower positive end-expiratory pressures in patients with the acute respiratory distress syndrome. N Engl J Med. (2004) 351:327–36. doi: 10.1056/NEJMoa032193 15269312

[11] Guérin C Reignier J Richard JC Beuret P Gacouin A Boulain T. Prone positioning in severe acute respiratory distress syndrome. N Engl J Med. (2013) 368:2159–68. doi: 10.1056/NEJMoa1214103 23688302

[12] Amato MB Meade MO Slutsky AS Brochard L Costa EL Schoenfeld DA. Driving pressure and survival in the acute respiratory distress syndrome. N Engl J Med. (2015) 372:747–55. doi: 10.1056/NEJMsa1410639 25693014

[13] Cressoni M Gotti M Chiurazzi C Massari D Algieri I Amini M. Mechanical power and development of ventilator-induced lung injury. Anesthesiology. (2016) 124:1100–8. doi: 10.1097/aln.0000000000001056 26872367

[14] Beitler JR Sarge T Banner-Goodspeed VM Gong MN Cook D Novack V. Effect of titrating positive end-expiratory pressure (PEEP) with an esophageal pressure-guided strategy vs an empirical high PEEP-Fio2 strategy on death and days free from mechanical ventilation among patients with acute respiratory distress syndrome: a randomized clinical trial. JAMA. (2019) 321:846–57. doi: 10.1001/jama.2019.0555, PMID: 30776290 PMC6439595

[15] Chen L Chen GQ Shore K Shklar O Martins C Devenyi B. Implementing a bedside assessment of respiratory mechanics in patients with acute respiratory distress syndrome. Crit Care. (2017) 21:84. doi: 10.1186/s13054-017-1671-8, PMID: 28372575 PMC5379641

[16] Grasselli G Calfee CS Camporota L Poole D Amato MBP Antonelli M. ESICM guidelines on acute respiratory distress syndrome: definition, phenotyping and respiratory support strategies. Intensive Care Med. (2023) 49:727–59. doi: 10.1007/s00134-023-07050-7, PMID: 37326646 PMC10354163

[17] Eysenbach G. Welcome to the journal of medical internet research. J Med Internet Res. (1999) 1:e5. doi: 10.2196/jmir.1.1.e5

[18] Cooper ID. Bibliometrics basics. J Med Libr Assoc. (2015) 103:217–8. doi: 10.3163/1536-5050.103.4.013, PMID: 26512226 PMC4613387

[19] He Z Dai L Zuo Y Chen Y Wang H Zeng H. Hotspots and frontiers in pulmonary arterial hypertension research: a bibliometric and visualization analysis from 2011 to 2020. Bioengineered. (2022) 13:14667–80. doi: 10.1080/21655979.2022.2100064, PMID: 35880647 PMC9342150

[20] Lin GX Nan JN Chen KT Sun LW Tai CT Jhang SW. Bibliometric analysis and visualization of research trends on oblique lumbar interbody fusion surgery. Int Orthop. (2022) 46:1597–608. doi: 10.1007/s00264-022-05316-1, PMID: 35099577

[21] Yao RQ Ren C Wang JN Wu GS Zhu XM Xia ZF. Publication trends of research on Sepsis and host immune response during 1999-2019: a 20-year bibliometric analysis. Int J Biol Sci. (2020) 16:27–37. doi: 10.7150/ijbs.37496, PMID: 31892843 PMC6930382

[22] Sweileh WM. Patient satisfaction with nursing care: a bibliometric and visualization analysis (1950-2021). Int J Nurs Pract. (2022) 28:e13076. doi: 10.1111/ijn.13076 35822232

[23] Khan FR Raza Kazmi SM Siddiqui YF. A bibliometric analysis of the studies on dental implant failure. J Pak Med Assoc. (2022) 72:S76–s80. doi: 10.47391/jpma.Aku-15 35202375

[24] YildirimFGulhanPYKaramanIKurutkanMN. Bibliometric analysis of acute respiratory distress syndrome (ARDS) studies published between 1980 and 2020. Adv Clin Exp Med. (2022) 31:807–13. doi: 10.17219/acem/150555, PMID: 35699587

[25] Zhang X Wang C Zhao H. A bibliometric analysis of acute respiratory distress syndrome (ARDS) research from 2010 to 2019. Ann Palliat Med. (2021) 10:3750–62. doi: 10.21037/apm-20-2050, PMID: 33752427

[26] Wang C Wang X Long X Xia D Ben D Wang Y. Publication trends of research on acute lung injury and acute respiration distress syndrome during 2009-2019: a 10-year bibliometric analysis. Am J Transl Res. (2020) 12:6366–80. PMID: 33194036 PMC7653614

[27] Aria M Cuccurullo C. Bibliometrix: an R-tool for comprehensive science mapping analysis. J Informet. (2017) 11:959–75. doi: 10.1016/j.joi.2017.08.007

[28] Van Eck NJ Waltman L. Software survey: VOSviewer, a computer program for bibliometric mapping. Scientometrics. (2010) 84:523–38. doi: 10.1007/s11192-009-0146-3, PMID: 20585380 PMC2883932

[29] Wu Z McGoogan JM. Characteristics of and important lessons from the coronavirus disease 2019 (COVID-19) outbreak in China: summary of a report of 72 314 cases from the Chinese Center for Disease Control and Prevention. JAMA. (2020) 323:1239–42. doi: 10.1001/jama.2020.2648, PMID: 32091533

[30] Fan J Hambly BD Bao S. The epidemiology of COVID-19 in the Gansu and Jinlin provinces. China Front Public Health. (2020) 8:555550. doi: 10.3389/fpubh.2020.555550, PMID: 33042952 PMC7517784

[31] Ranieri VM Rubenfeld G Slutsky AS. Rethinking ARDS after COVID-19. If a “better” definition is the answer, what is the question? Am J Respir Crit Care Med. (2022) 207:255–60. doi: 10.1164/rccm.202206-1048CP, PMID: 36150099 PMC9896638

[32] Bhatt A Deshwal H Luoma K Fenianos M Hena K Chitkara N. Respiratory mechanics and association with inflammation in COVID-19-related ARDS. Respir Care. (2021) 66:1673–83. doi: 10.4187/respcare.09156 34521759

[33] Somhorst P Gommers D Endeman H. Advanced respiratory monitoring in mechanically ventilated patients with coronavirus disease 2019-associated acute respiratory distress syndrome. Curr Opin Crit Care. (2022) 28:66–73. doi: 10.1097/mcc.0000000000000905, PMID: 34772836 PMC8711301

[34] Vandenbunder B Ehrmann S Piagnerelli M Sauneuf B Serck N Soumagne T. Static compliance of the respiratory system in COVID-19 related ARDS: an international multicenter study. Crit Care. (2021) 25:52. doi: 10.1186/s13054-020-03433-0, PMID: 33557868 PMC7868865

[35] Gattinoni L Pelosi P Suter PM Pedoto A Vercesi P Lissoni A. Acute respiratory distress syndrome caused by pulmonary and extrapulmonary disease. Different syndromes? Am J Respir Crit Care Med. (1998) 158:3–11. doi: 10.1164/ajrccm.158.1.9708031 9655699

[36] Chiumello D Carlesso E Cadringher P Caironi P Valenza F Polli F. Lung stress and strain during mechanical ventilation for acute respiratory distress syndrome. Am J Respir Crit Care Med. (2008) 178:346–55. doi: 10.1164/rccm.200710-1589OC 18451319

[37] Liu J Zhang L Ma P. A new era of critical care research in China. J Crit Care. (2019) 54:20–1. doi: 10.1016/j.jcrc.2019.07.005, PMID: 31325814

[38] Pelosi P Tubiolo D Mascheroni D Vicardi P Crotti S Valenza F. Effects of the prone position on respiratory mechanics and gas exchange during acute lung injury. Am J Respir Crit Care Med. (1998) 157:387–93. doi: 10.1164/ajrccm.157.2.97-04023 9476848

[39] Pelosi P Brazzi L Gattinoni L. Prone position in acute respiratory distress syndrome. Eur Respir J. (2002) 20:1017–28. doi: 10.1183/09031936.02.00401702 12412699

[40] Vieira ES. International research collaboration in Africa: a bibliometric and thematic analysis. Scientometrics. (2022) 127:2747–72. doi: 10.1007/s11192-022-04349-y

[41] Pelosi P Ball L Barbas CSV Bellomo R Burns KEA Einav S. Personalized mechanical ventilation in acute respiratory distress syndrome. Crit Care. (2021) 25:250. doi: 10.1186/s13054-021-03686-3 34271958 PMC8284184

[42] Matthay MA Arabi YM Siegel ER Ware LB Bos LDJ Sinha P. Phenotypes and personalized medicine in the acute respiratory distress syndrome. Intensive Care Med. (2020) 46:2136–52. doi: 10.1007/s00134-020-06296-9, PMID: 33206201 PMC7673253

