# Supplementary material for: A bibliometric analysis of respiratory mechanics research related to acute respiratory distress syndrome from 1985 to 2023
Source: Front Med (Lausanne). 2024 Sep 20;11:1420875. doi: 10.3389/fmed.2024.1420875 (PMC11449829; doi:10.3389/fmed.2024.1420875)
Supplement: Supplementary file 1 [file Table_1.DOCX]

**Supplementary Table 1**. The analytic consequence of 77 keywords with at least 30 occurrence times

| No. | Label | Cluster | Links | Occurrences | Average publication years | Average citations |
| --- | --- | --- | --- | --- | --- | --- |
| 1 | mechanical ventilation | 3 | 76 | 519 | 2012.88 | 32.14 |
| 2 | acute lung injury | 2 | 76 | 474 | 2011.39 | 36.91 |
| 3 | respiratory-distress-syndrome | 2 | 72 | 467 | 2011.50 | 32.64 |
| 4 | end-expiratory pressure | 2 | 76 | 421 | 2010.85 | 37.51 |
| 5 | acute respiratory distress syndrome | 3 | 73 | 355 | 2013.62 | 31.37 |
| 6 | ards | 3 | 76 | 347 | 2013.28 | 27.66 |
| 7 | ventilation | 1 | 76 | 240 | 2011.62 | 34.68 |
| 8 | respiratory mechanics | 3 | 75 | 224 | 2012.65 | 29.00 |
| 9 | failure | 1 | 74 | 193 | 2006.38 | 37.60 |
| 10 | recruitment | 1 | 73 | 192 | 2011.35 | 31.32 |
| 11 | mechanics | 1 | 75 | 188 | 2009.44 | 31.57 |
| 12 | gas-exchange | 1 | 72 | 160 | 2007.76 | 34.32 |
| 13 | injury | 1 | 74 | 149 | 2012.88 | 32.14 |
| 14 | tidal volume | 2 | 71 | 140 | 2009.30 | 43.82 |
| 15 | distress-syndrome | 3 | 68 | 128 | 2013.36 | 29.22 |
| 16 | model | 1 | 74 | 114 | 2008.99 | 27.93 |
| 17 | pressure | 1 | 70 | 112 | 2012.71 | 25.02 |
| 18 | oxygenation | 1 | 72 | 106 | 2009.20 | 36.19 |
| 19 | mortality | 3 | 68 | 105 | 2016.26 | 23.80 |
| 20 | pulmonary | 1 | 72 | 101 | 2010.01 | 34.00 |
| 21 | peep | 3 | 70 | 101 | 2011.15 | 29.25 |
| 22 | positive end-expiratory pressure | 3 | 74 | 98 | 2012.42 | 31.96 |
| 23 | lung injury | 3 | 71 | 94 | 2012.48 | 21.62 |
| 24 | covid-19 | 3 | 56 | 92 | 2021.64 | 16.63 |
| 25 | chest-wall mechanics | 2 | 61 | 91 | 2009.29 | 50.31 |
| 26 | ventilator-induced lung injury | 2 | 69 | 91 | 2014.18 | 39.21 |
| 27 | volume | 1 | 69 | 90 | 2009.30 | 31.76 |
| 28 | system | 1 | 67 | 88 | 2006.30 | 47.11 |
| 29 | respiratory-distress syndrome | 1 | 59 | 86 | 1998.90 | 42.90 |
| 30 | alveolar recruitment | 2 | 70 | 80 | 2008.23 | 49.61 |
| 31 | transpulmonary pressure | 3 | 68 | 78 | 2017.19 | 34.21 |
| 32 | prone position | 3 | 64 | 77 | 2013.79 | 30.64 |
| 33 | lung | 1 | 63 | 72 | 2009.56 | 27.83 |
| 34 | esophageal pressure | 3 | 62 | 72 | 2016.64 | 24.53 |
| 35 | airway pressure | 2 | 66 | 68 | 2009.71 | 43.96 |
| 36 | lung mechanics | 1 | 65 | 64 | 2011.30 | 28.69 |
| 37 | pulmonary-edema | 2 | 65 | 60 | 2008.35 | 60.85 |
| 38 | strategy | 2 | 63 | 60 | 2012.12 | 37.32 |
| 39 | driving pressure | 3 | 65 | 57 | 2020.32 | 17.91 |
| 40 | recruitment maneuvers | 2 | 64 | 55 | 2013.84 | 35.35 |
| 41 | inflammation | 1 | 55 | 52 | 2012.77 | 29.23 |
| 42 | surfactant | 1 | 55 | 49 | 2006.80 | 28.63 |
| 43 | resistance | 1 | 52 | 47 | 2005.83 | 24.87 |
| 44 | respiratory distress syndrome | 1 | 57 | 45 | 2010.22 | 22.27 |
| 45 | computed-tomography | 2 | 59 | 45 | 2014.29 | 35.31 |
| 46 | tidal volumes | 2 | 59 | 44 | 2014.34 | 21.95 |
| 47 | induced lung injury | 3 | 61 | 44 | 2012.23 | 28.45 |
| 48 | derecruitment | 2 | 53 | 42 | 2009.86 | 48.69 |
| 49 | flow | 1 | 56 | 41 | 2009.37 | 22.41 |
| 50 | lower inflection point | 2 | 56 | 41 | 2006.37 | 39.05 |
| 51 | therapy | 1 | 48 | 40 | 2009.10 | 29.13 |
| 52 | protective-ventilation | 2 | 58 | 40 | 2011.45 | 26.73 |
| 53 | pulmonary mechanics | 1 | 61 | 38 | 2008.16 | 30.92 |
| 54 | gas exchange | 1 | 61 | 37 | 2007.89 | 27.49 |
| 55 | lung compliance | 1 | 53 | 37 | 2008.62 | 30.89 |
| 56 | chest-wall | 2 | 58 | 36 | 2006.42 | 59.72 |
| 57 | survival | 3 | 51 | 36 | 2016.50 | 15.36 |
| 58 | children | 1 | 48 | 35 | 2009.43 | 24.91 |
| 59 | randomized controlled-trial | 2 | 53 | 35 | 2012.74 | 39.11 |
| 60 | electrical impedance tomography | 3 | 53 | 35 | 2016.94 | 37.06 |
| 61 | regional-distribution | 2 | 58 | 34 | 2010.35 | 40.21 |
| 62 | general-anesthesia | 2 | 55 | 33 | 2010.00 | 48.67 |
| 63 | acute respiratory-distress | 3 | 60 | 33 | 2015.24 | 24.39 |
| 64 | protective ventilation | 3 | 60 | 33 | 2017.58 | 35.27 |
| 65 | partial liquid ventilation | 1 | 41 | 32 | 2003.53 | 20.03 |
| 66 | perfusion | 1 | 50 | 32 | 2011.94 | 28.19 |
| 67 | extracorporeal membrane-oxygenation | 3 | 49 | 32 | 2015.13 | 34.59 |
| 68 | management | 3 | 44 | 32 | 2014.53 | 19.06 |
| 69 | atelectasis | 1 | 52 | 31 | 2012.74 | 26.71 |
| 70 | expression | 1 | 43 | 31 | 2011.19 | 31.16 |
| 71 | pulmonary surfactant | 1 | 39 | 31 | 2007.81 | 37.45 |
| 72 | acute respiratory distress syndrome (ards) | 3 | 54 | 31 | 2015.71 | 17.84 |
| 73 | distress syndrome | 1 | 38 | 30 | 2000.00 | 31.50 |
| 74 | electrical-impedance tomography | 2 | 57 | 30 | 2016.03 | 28.30 |
| 75 | recruitment maneuver | 2 | 55 | 30 | 2012.20 | 32.57 |
| 76 | tidal volume ventilation | 2 | 52 | 30 | 2011.33 | 32.17 |
| 77 | respiratory failure | 3 | 50 | 30 | 2012.67 | 33.87 |

Abbreviations *ARDS*: Acute Respiratory Distress Syndrome; *PEEP*: Positive End-expiratory Pressure; *COVID-19*: Coronavirus Disease 2019.
